# Supplementary material for: Chromothripsis during telomere crisis is independent of NHEJ, and consistent with a replicative origin
Source: Genome Res. 2019 May;29(5):737–49. doi: 10.1101/gr.240705.118 (PMC6499312; doi:10.1101/gr.240705.118)
Supplement: Supplemental Material [file supp_gr.240705.118_Supplemental_file_1.zip › contigs/annotated_contigs/DB78/contig.2.DB78_length_629_mean_cov_5.86645468998.docx]

**DB78_length_629_mean_cov_5.86645468998**

```
TAGGTCATAAACTAAAATATTTAAAATTTATACTTTATTGCTAAAGTTAGTAATATCCATATTACTAACTAGCCAAATTATGAGAAAGG
 >chr5:166368867-166369198 - E=1e-173 p=8e-03
CTGAAGAAAACTTTAATACTTACAGCATTAATTTTACTGAAACTGTCTAATCTAGTAACTAATATTAATATTCAAACAATGTAAAAAAA

TTATTTTATTTATTTTTTTTGAGACGAAGTCTCGCTCTTGTCCCCCAGTCTGGGGTGCAATGGCGCAATCTCAGCTCACTGCAACCTCT

GCCTCCCGGGTTCAAGCGAGTCTCCTGCCTCAGCCTCCCGAGTAGCTGGGATTACAG|G|AATCTGAGAAAGTAAAAAACATCAAAGAA
                                                          >chr5:166367973-166368278 - E
TGCTACTTTACATGAATATCGTACAAAATACAGAACACCAGAAAATATGGATATCGGAAGTCTCCTTTTGCTATCCATGGTGAAGAAGC
=8e-169
CATTTTTACGATCTCAGCTCACCTGGGTAACCCTAACCCTAACCCTAATCCTACACCTTTGAAACAAGCTAAGCATGTTTCGCTGCTCA

CACTCAATGGATTCTACTGGCTTCATAAGGATAAAATGCTCTGAATAGGATGTATCTTGATCTTCTCTAGGTAACTTTAAGATGCATAG

GAGTTCAT
```
